# Evaluation of the Virtual Economic Effect of Tourism Product Emotional Marketing Based on Virtual Reality

**DOI:** 10.3389/fpsyg.2021.759268

**Published:** 2021-11-25

**Authors:** Manati Talafubieke, Sheng Mai, Nuermila Xialifuhan

**Affiliations:** School of Economics and Management, Shihezi University, Shihezi, China

**Keywords:** virtual reality, tourism products, emotional marketing, economic effect evaluation, consumption structure

## Abstract

Rapid development of Internet technology has led to great changes in all fields of life. Tourism industry has also gone to the Internet with the change of the times. Regarding the application of Internet technology in tourism, in fact, virtual tourism products are directly used to promote economic development. Online tourism stores have all kinds of tourism information in the database, realize network roaming, provide convenient information services for both the supply and demand of tourism, and can realize the rapid and stable sustainable development of tourism. This article mainly studies the evaluation of the virtual economic effect of emotional marketing of tourism products based on virtual reality (VR), the meaning algorithm of VR, etc., analyzes the virtual economy of tourism products based on VR, and helps tourism enterprises to grasp the needs of tourism consumers more accurately, so as to design products and services that are more in line with the demands of tourism consumers, the process of ultimately improving the user value. This study mainly uses the VR method, VR platform eye map processing, VR eye map regional detection and feature extraction method, and VR tourism product emotional marketing related experiments, to study the evaluation of the virtual economic effect of tourism product emotional marketing based on VR. The economic performance information of the virtual tourism industry is equivalent to the amount of these indicators, and the impact of the comprehensive evaluation on the economic performance of virtual tourism is quite equal. The results show that from 2017 to 2020, the virtual economic revenue of VR tourism product emotional marketing increased from 50 million to 1 billion. However, people give more importance to the tourism experience brought by virtual tourism products, and the reasonable consumption structure of virtual tourism hinders the improvement of tourism economic benefits. To promote the rationalization of the consumption structure of virtual tourism, the basic industry of tourism consumption is developed and the necessary design and processing is increased, in order to increase the supply of tourism products and to enrich tourism consumption.

## Introduction

With the rapid development of computer technology and Internet technology, the network is indispensable in the national life. The progress of science and technology has greatly changed the daily life of people. Therefore, it is very important to explore the possibility of network development. Virtual reality (VR) is a technology that uses computers and other hardware devices to simulate the three-dimensional world. Ideal VR can mobilize five officials of human beings and immerse people in VR.

To develop the national economy and improve the living standard of people, people will pay more attention to the development of the spiritual world. Tourism is one of the important events in holiday life, and the deputy director of China National Administration Report Bureau and the vice president of China Report Association said that tourism has become the most reasonable and potential field to promote economic growth, which also provides great development potential for new forms of real tourism products. The current tourism products cannot provide real virtual displays and structured virtual services for tourists. With the continuous progress of VR technology and yearning for VR technology of tourists, the current tourism products cannot meet the needs of tourism consumers in terms of interaction, immersion, and authenticity. Thus, VR technology needs to be applied to the field of Internet tourism. We will continue to promote the healthy development of internet tourism.

Virtual reality technology will gradually become one of the important technologies to promote the digitalization of tourism information in the future. The VR technology is applied to digital tourism technology to realize the VR of tourism products. Bagus et al. believe that the concept of wireless augmented VR has recently swept the entire 5G ecosystem, causing unprecedented interest in academia, industry and other fields (Bagus et al., 2020). However, the success of the VR experience in the present situation depends on solving a large number of major challenges across multiple disciplines. At the same time, it emphasizes the importance of VR technology as a destructive use case of 5G, which takes advantage of the latest development in storage/memory, fog/edge computing, computer vision, artificial intelligence, and so on. In particular, the main requirements of wireless interconnection VR are described, and then, the key enabling technology is chosen; the research approaches and potential major challenges are introduced. In addition, we have studied three VR cases, given the numerical results under different storages, calculations, and network configurations, also revealed the limitations of the current network, and put forward more theories and innovative reasons, which laid the groundwork for the development of the mass VR but lacked specific data before the project (Bastug et al., 2017). Riva et al. believed that it is possible to make significant changes in body image, attitude, and behavior of obese and related diseases using VR-based psychological methods. The design used is a clinical intervention study, which is treated five times a week based on VR. This improvement is always related to a reduction in problematic diet and social behavior. In traditional weight loss programs, the possibility of inducing significant changes in body image and related behaviors by using short-term VR-based therapy can improve physical satisfaction, but there is no necessary experimental data (Riva et al., 2016). Lenoir J believed that the potential of virtual tourism in Africa is a way to promote economic growth and sustainability under the influence of the coronavirus disease 2019 (COVID-19) epidemic, archives is engaged in the research of archival methods or literature review, and virtual tourism may benefit millions of people. The document helps to innovate strategies for integrated and regional virtual or online tourist attractions such as game parks, protected areas, and other tourist attractions in Africa. African governments must seriously consider establishing partnerships with the private sector to build virtual technology experiments for virtual interaction and enterprise resilience but lack numerical analysis contents (Lenoir et al., 2017). Elbamby et al. believed that VR is expected to be one of the killer applications of 5G network. However, many technical bottlenecks and challenges need to be overcome to promote their wide adoption. Especially, the demand of VR in high throughput, low latency, and reliable communication requires innovative solutions and basic research across multiple disciplines. The challenge and implementation method of super reliable and low delay VR are needed. In addition, in a case study of an interactive VR game machine, the experiment shows an intelligent network design. With the use of millimeter-wave communication, edge computing, and active cache, the future vision of VR can be realized, and it can be used for reference but some of the theories are not accurate (Elbamby et al., 2018).

The innovation of this study is to study the evaluation of the virtual economic effect of tourism product emotional marketing based on VR, the meaning algorithm of VR, and analyze the virtual economy of tourism products based on VR, so as to meet the daily needs of tourists. In the virtual tourism experience, we should pay more attention to beautifying the public and scenic area ecological environment, increasing the attraction of the city, and realizing the concept of “everywhere scenery” of the whole tourism (Freeman et al., 2017).

## VR Method

### Virtual Reality

**Figure 1:**
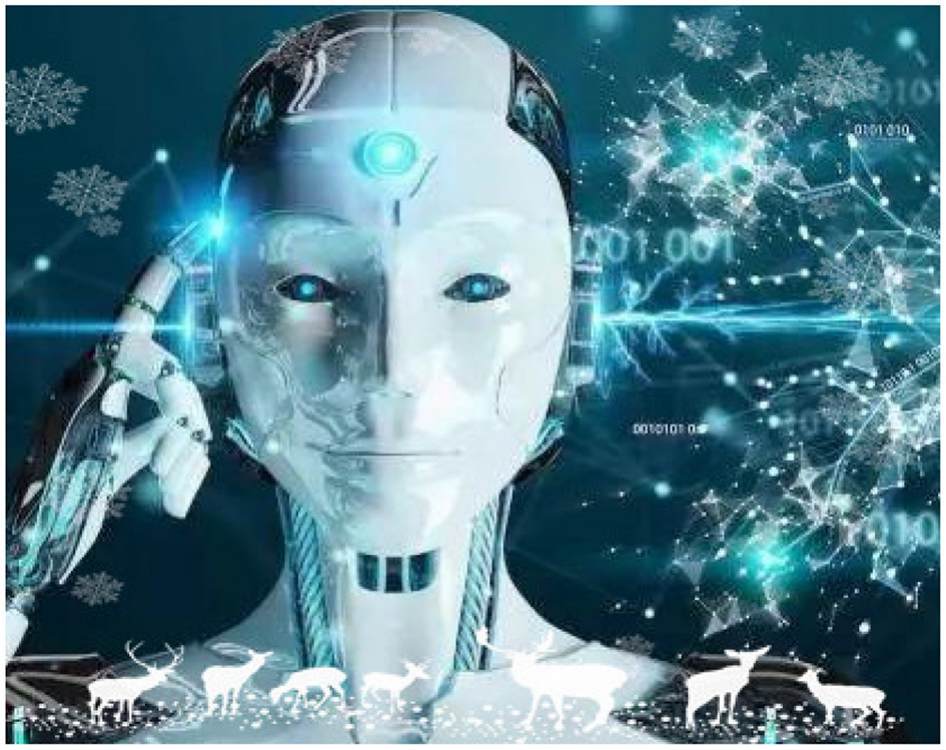
Virtual reality (VR) (http://alturl.com/boffn).

Virtual reality technology is to simulate a 3D fictional space for users by computer technology. It can simulate visual, tactile, auditory, and other sensory feelings and quickly observe real things. In the process of interaction, the computer uses its powerful computing power to transfer all kinds of information in the simulated three-dimensional virtual world to users, so as to provide the user with the experience of being in the real world (Freeman et al., 2016). With the rapid development of VR technology, more and more VR products are available in the market, but the natural interaction and dizziness in VR technology have not been solved well (Saposnik et al., 2016; Sharar et al., 2016). Therefore, the importance of eye-tracking technology in VR is self-evident. It can be used as a supplement to the human-computer interaction in VR, increase the immersion of users, and also provide immersion and enhanced experience. It has been widely used in various fields (Falconer et al., 2016). The application of VR in reality mainly includes aerospace and military, games and entertainment, and medical research. In recent years, technology has developed rapidly in entertainment games. VR technology can provide more real and rich perception for gamers, and let users experience 3D reality effects and the perception effect of the situation in movies and games as shown in Figure 1.

### Eye Image Processing of VR Platform

#### Methods of Pupil Location

Accurate pupil location is the key to obtain effective eye movement data. The common methods of pupil detection include circle detection, ellipse fitting, and CAMSHIFT algorithm. In image processing, Hough transform is a common method to detect circle area (Berg and Vance, 2017). From a mathematical point of view, the algebraic equation is used to represent the contour of the measured area, and the Hough transform method is used to define the standard equation of the circle, as shown in the formula:


(1) $$\begin{array}{r} {\left(x-m\right)}^{2}+{\left(y-n\right)}^{2}=r^{2} \end{array}$$

In the above formula, the coordinates (*m* and *n*) are used to represent the coordinates of the center of the circle, and the radius of the circle is expressed by *R*. In general, the pupil area can be regarded as a circle, so Hough transform can be used to detect the circle to identify the pupil area. It is a common method to fit the edge of the target region with the least square ellipse. This method is mature and suitable for the situation that the edge of the target region is close to the ellipse (Donath et al., 2016; Munafo et al., 2017). In Cartesian coordinates, the general form of the elliptic equation is as follows:


(2) $$\begin{array}{r} mx^{2}+nxy+ay^{2}+bx+cy=0 \end{array}$$

where *x* and *y* are the center coordinates of the ellipse, and *m* and *N* are the long and short half axes of the ellipse.


(3) $$\begin{array}{r} x=\left(nb-2ab\right)/\left(4ma-n^{2}\right) \end{array}$$

#### Non-linear Transformation

The pixel data in the circular lens area of the eye movement image are processed, and the gray value of the data in the area is transformed non-linearly. Some data information may be lost through the non-linear transformation of image gray, but the information that the gray value of the original image does not change significantly can be enhanced (Munafo et al., 2017). We can change the mapping relation of non-linear transformation to get different effects and obtain the required image information. The non-linear gray transformation of the image can be divided into non-linear logarithmic transformation and non-linear exponential transformation to lay a good foundation for logarithmic relation:


(4) $$\begin{array}{r} h\left(x,y\right)=\mathrm{lg}\left[h\left(x,y\right)\right] \end{array}$$

where (*x, y*) is the pixel coordinates in the image, *h*(*x, y*) is the gray value corresponding to the pixel coordinates, and *h*(*x, y*) is the gray value after logarithmic non-linear transformation of the gray value of the original coordinates. It is worth noting that one of the characteristics of the logarithmic function is that it can stretch the lower gray value of the image and compress the high gray area of the image (Vankipuram et al., 2017). Logarithmic change of image gray can expand the low gray area of the image, and the effect is better when the image is dark. Exponential non-linear transformation means that the gray value between the transformed image and the image before transformation satisfies the exponential relation:


(5) $$\begin{array}{r} h\left(x,y\right)=b^{h\left(x,y\right)} \end{array}$$

where *b* is the base of the function, (*x, y*) is the coordinates of the pixels in the image, *h*(*x, y*) is the coordinates of the pixels, the corresponding gray value, and *h*(*x, y*) refers to the gray value of the original coordinate point after logarithmic non-linear transformation. It should be pointed out that the exponential transform is mainly used to expand the high gray area, which can expand the gray value of the brighter pixels in the original image, and is generally suitable for the over bright image (Meilin, 2017).

#### Near IR Light Source

The radiation intensity of the near-IR light source is 350 MW/Sr, it is assumed that the exposure time of human eyes to near-IR light is much longer than 1,000 s, the distance between human eyes and near-IR light source is ~6 cm, therefore, the radiation intensity value at this time needs to be solved, and the calculation formula is as follows:


(6) $$\begin{array}{r} D=E/l^{2} \end{array}$$

where *D* refers to the illuminance of the radiation, *E* refers to the intensity of the radiation, and *l* refers to the distance between the near-IR light and the human eye.


(7) $$\begin{array}{r} h\left(x,y\right)=\left[h\left(x,y\right)*\left(500-h\left(x,y\right)\right)\right] \end{array}$$

The ultimate goal of the clustering algorithm is to obtain close and independent classes through continuous iteration. *k*-means clustering aims to divide into *k* classes, where *k* < *n*, so that the original data information can be expressed as follows:


(8) $$\begin{array}{r} k=\left\{k_{1},k_{2},\cdots k_{n}\right\} \end{array}$$

In other words, the goal is to find the minimum value of the mathematical model as follows:


(9) $$\begin{array}{l} \arg\min{\overset{n}{\underset{i=1}{\sum }}\underset{x\in k}{\sum }\|x-k_{i}\|}^{2} \end{array}$$

#### VR Technology

**Figure 2:**
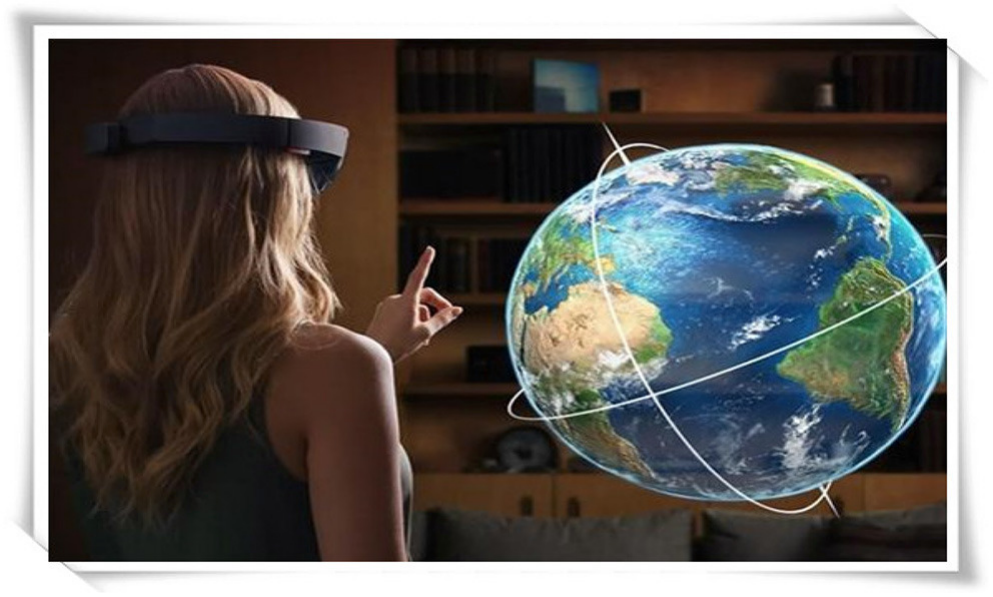
Application of VR technology (http://alturl.com/pxm5z).

Virtual reality technology is referred to as “VR.” VR technology is in the exploration period. At first, there were only some military and academic laboratory concepts. Until the beginning of this century, with the continuous development of computer and sensor technology, it gradually changed from the workplace to the practical stage and was widely used in different fields of human life, creating a new situation (Schmitz and Tsobgou, 2016). It realizes the natural communication between the user and the system, which not only improves the working efficiency of the system but also facilitates the operation of the user, as shown in Figure 2.

Virtual reality technology is to put the user into the virtual environment completely and realize the interaction with the electronic system using the natural perception of humans. Explicitly, with the own visual, auditory, tactile, and other senses of human beings, the user can get the same feeling of the visual, auditory, tactile, and other stimuli provided by the system in the system as in the real world (Bagus et al., 2020; Szczsna and Wesoowska, 2020).

#### Region Detection and Feature Extraction of VR Eye Map

The maximum stable extremal region detection is based on the idea of the watershed, which is described as continuous water injection into an area. With the increase of water, any gap in this area will narrow, but not all areas will rise underwater at the same time but by the way of water overflow. When the two regions are connected, the water surface of the two regions increases at the same time, but in this process, the increase of water surface height will not change significantly in some regions (Lan, 2020). The concept of drainage is used to analyze the basic image of eye movement. In this process, some areas of the image will not change significantly or even remain unchanged when the scaling threshold is increased. Then, these regions are regarded as the maximum stable regions, and their mathematical definitions are as follows:


(10) $$\begin{array}{r} F\left(i\right)=|F_{i}+\Delta -F_{i}|/|F_{i}| \end{array}$$

where *F* represents one of the connected regions whose binarization threshold is I, represents the change of gray value, and obviously represents the change rate of connected region *f* (I) with I. For each pixel in the detected stable region, the calculation formula is as follows:


(11) $$\begin{array}{r} h=\sum I\left(x,y\right) \end{array}$$

After calculating the center of gravity of the whole region, that is the center of gravity of the ellipse (Oladipo and Modupe, 2020), then, we need to solve the major axis and minor axis of the ellipse as follows:


(12) $$\begin{array}{r} m=\sum {\left(x,y\right)}^{2}I\left(x,y\right) \end{array}$$

The gray level histogram is a very useful tool in digital image processing. It mainly reflects the content of the image gray level. The gray level information is very important for any image. Gray level histogram statistics refers to the number of gray level pixels in an image or the accuracy of gray level pixels. The structure of the one-dimensional gray level histogram is as follows:


(13) $$\begin{array}{r} F\left(n\right)=\left[m\left(x_{1}\right),m\left(x_{2}\right),\cdots m\left(x_{n}\right)\right] \end{array}$$

where *f* (*x*) is the number of pixels, *m*(*x*) is the total number of pixels, in the case of image gray histogram, and the abscissa is gray. The ordinate is the frequency of gray scale, and it can also be the number of pixels to calculate the gray scale value. Then, we analyzed the gray scale histogram of the intercepted eye image as follows:


(14) $$\begin{array}{r} m\left(x_{i}\right)=\frac{f\left(x_{i}\right)}{\sum f\left(x_{i}\right)} \end{array}$$

If the calculated fixation angular rate is greater than the given threshold, the eye motion mode of the frame is set to saccade, and there is no need to calculate the line of sight. Otherwise, it is fixation. To calculate the line of sight point, the variance of the angular velocity of fixation point needs to be calculated, as shown in the formula:


(15) $$\begin{array}{l} \sigma =\frac{1}{n}\sqrt{\sum_{i=0}^{n-1}(}f_{n-i}{\;}^{2}-f^{-2}) \end{array}$$

## Experiments on Emotional Marketing of Tourism Products Based on VR

### Function of Emotional Marketing

To meet the emotional needs of consumers is the core of emotional marketing. It is a process to stimulate the inner emotional needs of consumers by stimulating and meeting the emotional needs of consumers and to achieve marketing goals. Emotions of consumers can be divided into two different types, one is the result of daily accumulation and the other is a short-term attitude, whether positive or negative, calm or strong, can be classified into the emotional category. At present, society has huge material wealth, fierce competition, and increasingly personalized consumption. Emotional factors become important and special factors in the marketing process (Fabisiak, 2018). Enterprises have to create a positive emotional experience for consumers, for example, to make other consumers feel happy, happy and excited, so as to avoid their negative emotions. Emotion is the key factor of modern marketing. To obtain the recognition and loyalty of consumers, enterprises must connect consumers with emotion, that is, enterprises must pay attention to the emotional connection between consumers and brands, and emotional marketing emphasizes an enterprising spirit. To meet the emotional needs of consumers and make them feel psychological identity, consumers have a preference for corporate brands. Brand does not need the formation of consumer psychology (Khatwani and Srivastava, 2018).

### Tourism Product Module Function of VR

Virtual reality tourism products adopt the VR technology relied on the combination of graphics and images and adopt different development technologies for different modules, which not only saves the development cost to the greatest extent but also enhances the performance of VR tourism products. In this system, visitors can not only watch and listen when walking but also show the real environment and facilities of the hotel in front of tourists. In addition, tourists can fully integrate into the VR world through games and immerse themselves in the world of VR tourism products. It provides the possibility to develop different types of VR tourism products, which can meet different types of tourist experiences as much as possible. The tourism product module of VR is shown in Table 1.

**Table 1:** Tourism product function module of virtual reality (VR).

**Serial number**	**Modular**
1	Information inquiry and reservation
2	Online shopping
3	Virtual community
4	GIS
5	Virtual tourism

Virtual tourism products not only improve the tourism experience of VR tourism products but also help to improve the tourism experience of real tourists. The main information source of this survey is a questionnaire survey, which is based on the use of tourism products and the consumption of tourism products by ordinary users. This study studies the impact of the use of tourism products on brand acceptance, cognition, attitude, and behavioral change of users. The purpose is to measure the effect of brand communication of tourism products from the perspective of users and investigate the development of virtual tourism products in recent years, as shown in Table 2.

**Table 2:** Development of virtual tourism.

**Year**	**Total revenue of virtual tourism products**	**Total revenue growth rate**	**Total number of virtual tourism products**	**Total number growth rate**
2016	26,456	15.7%	15.6	9.5%
2017	3,47,666	18.5%	14.5	8.4%
2018	4,21,562	20.6%	12.4	6.6%
2019	2,53,567	14.4%	11.4	5.6%
2020	1,45,678	5.6%	17.6	10.2%

### Emotional VR Model of Virtual Tourism Products

**Figure 3:**
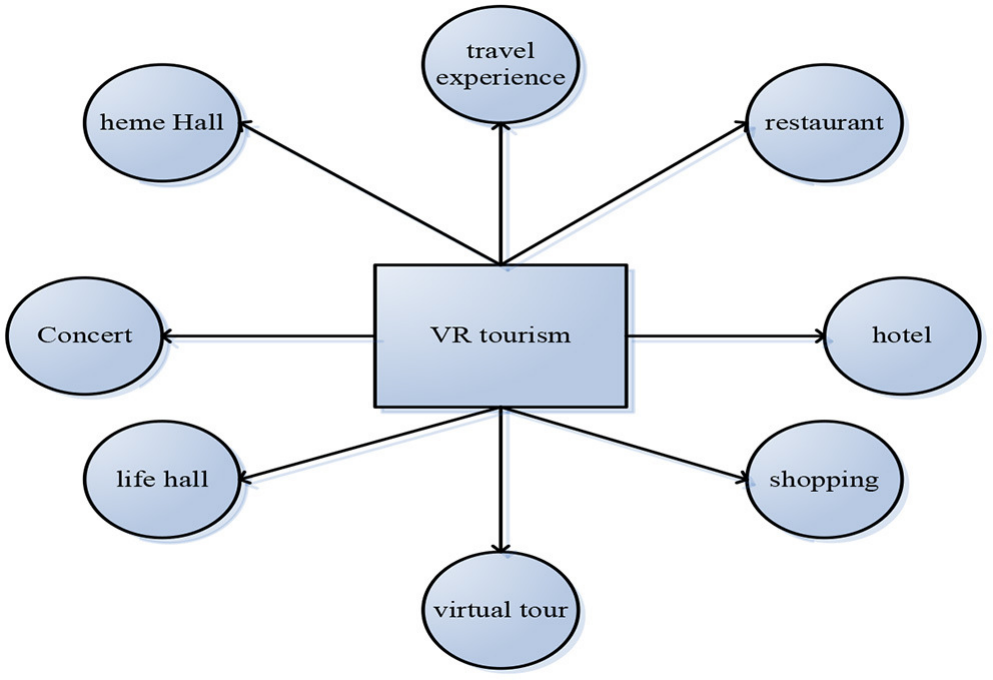
Virtual tourism emotional VR model.

The process of the research model is that based on the understanding of the connotation and dimension of the interaction between users in the tourism virtual community, we can understand the experience projects brought by the VR tourism products to the users. Then, according to the main elements of the theory of value co-creation and the context of its impact, this study constructs the research model and discusses and verifies the role of community users and online tourism enterprises in the interaction of virtual community users in tourism. Finally, based on the previous theories, this study verifies the influence of interaction and purchase decision, customer value and purchase decision, and verifies the regulatory effect of enterprise control on the model (Peng et al., 2019). First, the practical application of virtual community in the tourism industry is tourism virtual community, its main characteristics are the same as a virtual community, different from that of participants in tourism virtual community, the common interest among participants is tourism products and information, and the formation of virtual tourism emotional VR model is shown in Figure 3.

### Virtual Tourism

In recent years, during the small and long holidays, the news of overcrowding in popular tourist destinations at home and abroad occupies the headlines of the major media. In the case of such a large number of holiday tourists, it is particularly important to divert the number of tourists from popular tourist destinations by means of time shifting, traffic shifting, and choosing non-popular tourist routes. In addition to the above diversion methods, the virtual tourism developed by computer and Internet technology will also provide a new tourism mode for tourists to choose. Virtual tourism refers to the process that tourists participating in virtual tourism can personally carry out tourism activities by simulating or surpassing the real tourism landscape on the basis of the real tourism landscape, which can be divided into broad sense and narrow sense. In a broad sense, virtual tourism is closely related to traditional tourism, which mainly refers to the process of tourists obtaining relevant information of tourism destination before travel, such as through traditional and new media channels such as books, newspapers, television, and Internet. From the perspective of virtual tourism in a broad sense, virtual tourism and traditional tourism are simultaneous activities. The narrow sense of virtual tourism is more closely related to computer and Internet technology, mainly refers to the use of a variety of imaging technology VR tourism, and the process of making tourists get information about the tourist destination.

## Evaluation of Virtual Economy of Emotional Marketing of Tourism Products

### Virtual Tourism Plays Different Roles in the Six Types of Tourism Destination Development

The life cycle of a tourism destination is generally divided into six stages, namely, exploration period, participation period, development period, integration period, stagnation period, and recession period or recovery period. The development of the tourism industry in China is quite usual. According to Butler's tourism destination life cycle theory, the development of domestic tourism destinations can be divided into natural tourism destination, basic tourism destination, development tourism destination, popular tourism destination, tourism destination in decline period and tourism destination in recovery period as shown in Table 3.

**Table 3:** Six stages of tourism destination life cycle theory and its application.

**Sequence number**	**Life cycle**	
1	Exploration period	Natural tourism destination
2	Participation period	Basic tourism destination
3	Development period	Development tourism destination
4	Consolidation period	Popular tourism destination
5	Stagnation period	Popular tourism destination
6	Decline and recovery period	Decline and recovery period

According to Table 3, the main virtual tourism products can be used to try to produce leading or diversion effects in popular tourism destinations and also try to stimulate different effects in the six types of tourism destination development stages:

(1) Promotion function: in the natural tourist destination, the promotion form of virtual tourism products and is selected to increase the original tourism resources.
  Industrial exhibition, to show the world the charm of natural tourism, plays a leading role in the real sense.
(2) Attraction: in the basic tourism destination, the advertising form of virtual tourism products is selected and put on the market to attract more tourists.
(3) Introduction function: in the development of tourism destinations, the introduction form of virtual tourism products and is selected to let tourists in many scenic spots.

Choose a closer destination in the future.

(4) Distributary function: in popular tourist destinations, we should pay attention to the sales and popularization of virtual tourism products and pay attention to saturated tourism in peak season.

The number of customers is divided.

**Figure 4:**
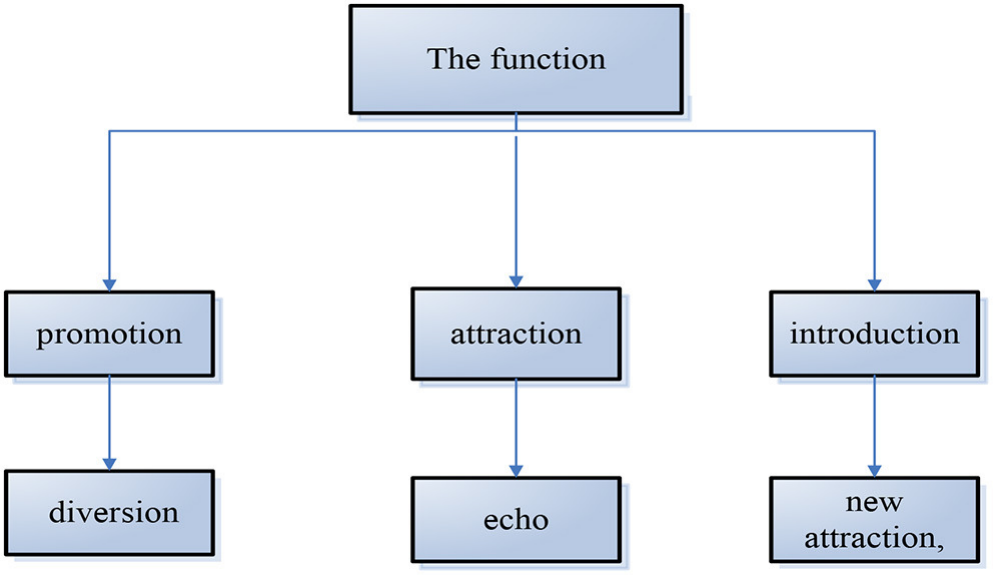
The function of virtual tourism products.

(5) Echo function: in the mature tourist destination, choose the propaganda form of virtual tourism products, to the tourists who have visited the tourist destination.
(6) New attraction: in declining or reviving tourist destinations, virtual tourism should be selected as a new attraction to arouse reconsideration of tourists and understanding of tourist destinations. Of course, virtual tourism products should play a good role in the six types of tourist destinations at the development stage, provided that policy makers should correctly understand and pay attention to the promotion of virtual tourism products, as shown in Figure 4.

Virtual tourism is a more convenient and relaxed choice. In addition to the good relationship between traditional tourism and virtual tourism, virtual tourism can also play different roles in the development of tourism destination.

### Advantages of Virtual Tourism Products

First, tourism products in tourism and emotional factors are culture, experience, and memory. In the construction of virtual tourism product scene, we can also put tourism and emotion-related factors into it for thinking and transform them into corresponding three designs of tourism emotion as follows: “cultural factors” form “cultural design,” “experience factors” form “interactive design,” and “memory factors” form “transformation design.” Guided by the three-level theory of emotional design, combined with the three-level design of tourism emotion, the “cultural factor” is transformed. For the level of “cultural design” corresponding to emotional “instinct design,” “experience factor” converted to “interactive design” corresponding to emotional “behavior design,” “memory factor” converted to “transformation design” corresponding to emotional “reflection design,” and the construction of virtual tourist attractions discussed in this study is shown in Table 4.

**Table 4:** Emotional design three-level theory.

**Serial number**	**Design**	**Factors**
1	Instinct design	Cultural factors
2	Behavior design	Interaction factors
3	Reflective design	Memory factors

It can be seen from Table 4 that the basic model framework of virtual tourism emotional design layer consists of three parts, namely, cultural design layer, emotional design layer, and transformation design layer, and “the cultural design” part is to use the cultural elements of virtual tourist attractions to awaken the recognition of tourists and appreciation of traditional tourist attractions. Considering the actual behavior needs of tourists in tourist attractions and the very important interactive part in virtual tourist attractions, the interactive function design reflects this, and the transformation design emphasizes the promotion of emotional sublimation of tourists in virtual tourism.

### Virtual Economic Effect of Virtual Tourism Products

With the rapid development of virtual tourism in recent years, the income of VR tourism products has been greatly improved. Its development should not be just a vague imitation, and specific development needs clear scientific planning and formulation including the comprehensive integration strategy of independent operation of provincial tourism bureaus and publicity of the overall image of national tourism bureaus. As a comprehensive and balanced tourism aggregate, China should formulate a comprehensive national tourism plan based on the specific development of local tourism characteristics, form differentiated tourism products, guide the complementary development of local tourism, and avoid fierce competition. Therefore, the quantitative analysis of China's VR tourism product income and the corresponding strategic suggestions are helpful to avoid the blindness and irrationality in the planning, form a scientific and reasonable industrial planning, reduce redundant construction and waste, strengthen the local cooperation and the overall collaborative development, improve the development efficiency, and strive to form a healthy and efficient industrial chain, According to the pattern of maximizing the internal and external economic and social benefits, the virtual tourism products, shops, and number of people experience in a county in recent 5 years are shown in Table 5.

**Table 5:** The economic effect of virtual tourism.

**Year**	**Virtual tourism product volume**	**Virtual tourism store**	**Number of virtual tourism experience**
2017	300	50	1,000
2018	500	80	5,000
2019	1,000	150	10,000
2020	5,000	400	70,000

**Figure 5:**
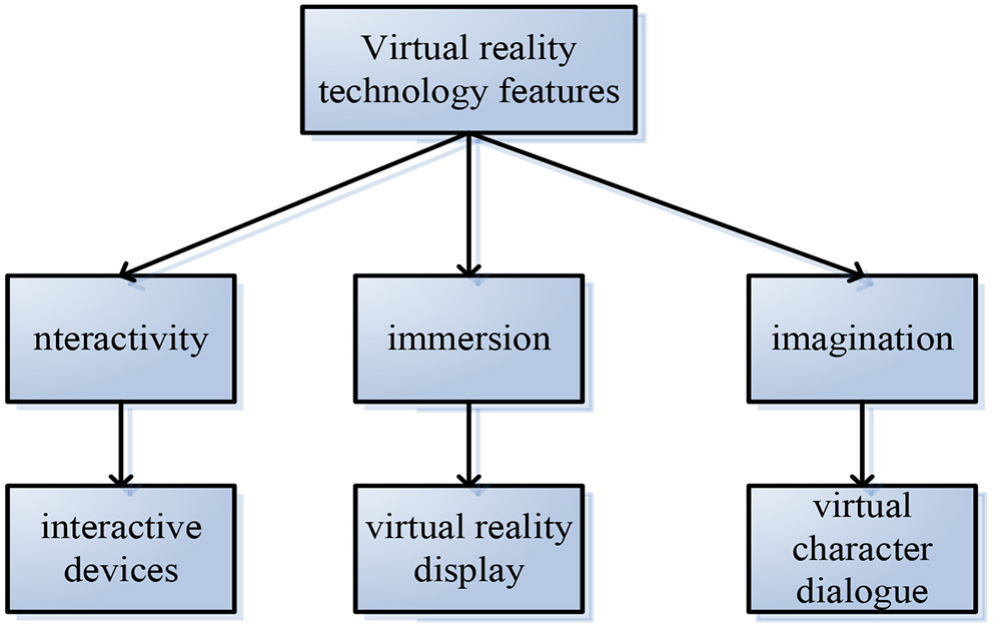
Characteristics of VR technology.

It can be seen from the table that with the increase of years from 2017 to 2020, the volume of virtual tourism products has increased from 300 to 5,000, the number of virtual tourism shops has increased from 50 to 400, and the number of virtual tourism experience has increased from 1,000 to 70,000. A three-dimensional virtual world can make people feel more real “reality” experience. The goal of combining VR technology with computer graphics, computer simulation, artificial intelligence, multimedia, sensors, and other technologies is to make the perception experience of people in the virtual world more and more delicate and real. The technical advantages of VR technology can enrich the design possibilities of more functions, experiences, and interfaces that can be added in the construction of virtual tourist attractions. The interactivity, immersion, and imagination of VR technology will stimulate more virtual experiences in future, as shown in Figure 5.

**Figure 6:**
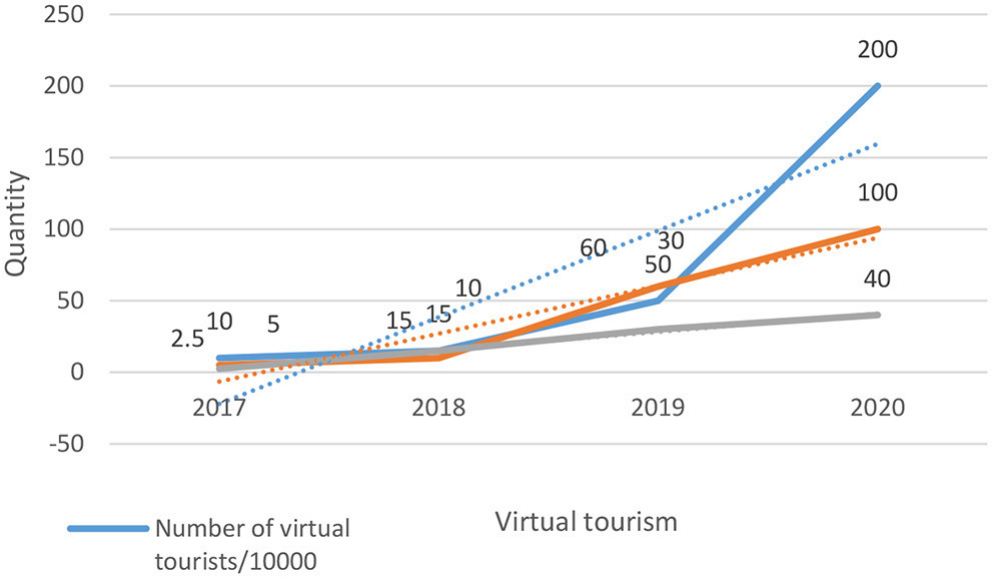
Parameter selection for different applications.

Although VR technology is designed based on the real world, its virtual nature can make the virtual world more imaginative and redesigning space than the real world. Designers add more or more interesting functions to the virtual user experience so that users can immediately feel the services that are difficult to have in reality and stimulate more imagination and thinking after experiencing VR products. From this point of view, VR will change the cognitive and learning styles of people. The economic effect of the VR tourism products investigated above is shown in Figure 6.

**Figure 7:**
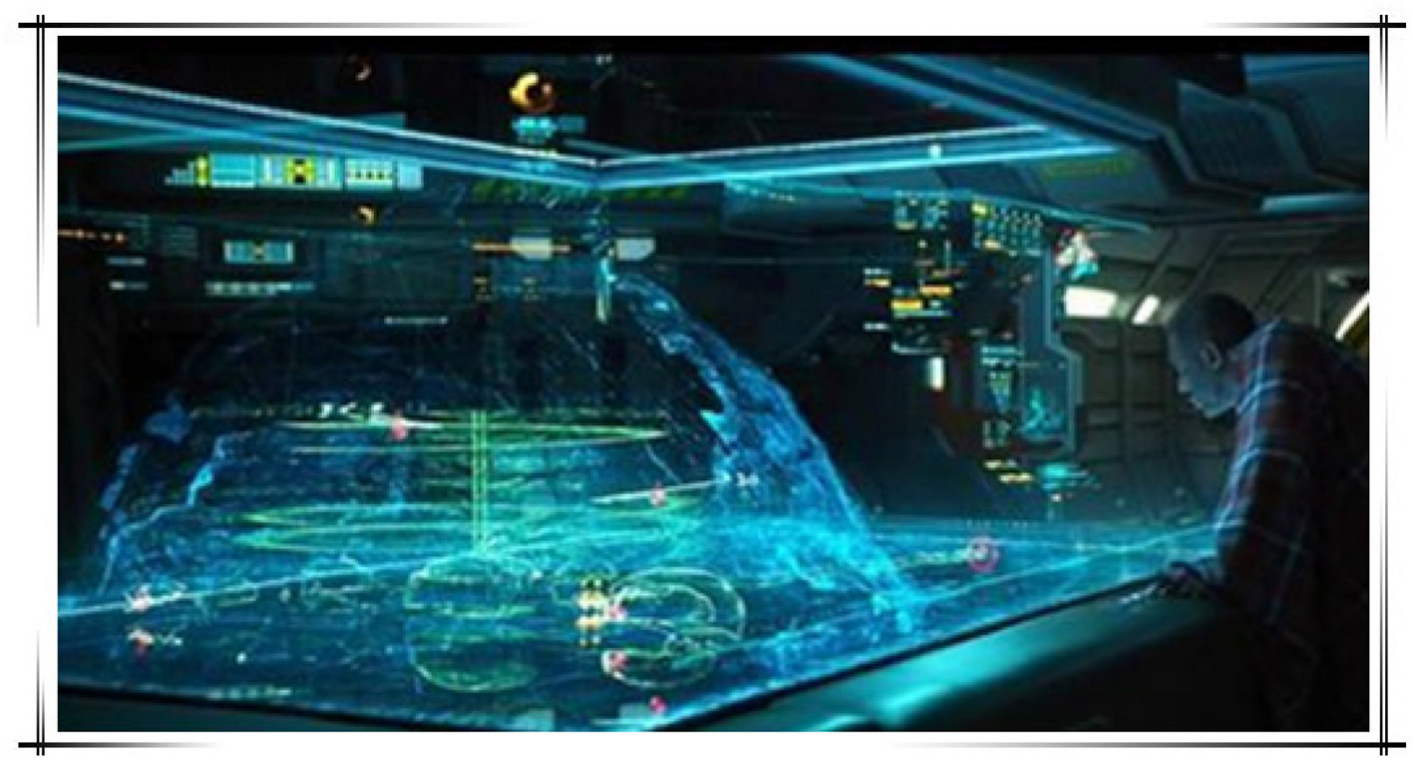
Technological innovation of information industry (http://alturl.com/knbeo).

It can be seen that from 2017 to 2020, the number of virtual tourists will increase from 1,00,000 to 2 million, the revenue of virtual products will increase from 50 million to 1 billion, and the growth rate will increase from 2.5 to 40%. In the VR tourism products, immersive VR strives to create an immersive environment for users, put the five senses of users in the VR space for natural interaction, use various output and input devices for interaction in line according to the law of movement, and pay attention to the experience of virtual simulation. This type of equipment has high requirements and strong experimental nature and is not easy to be popularized before the cost performance and portability of related equipment are improved. Augmented reality VR is a kind of VR based on reality. It solves different problems by superimposing virtual illusion on the real scene. In recent years, with the progress of software and hardware, the topic of VR and augmented reality is more and more discussed. People pay more attention to and look forward to how VR and augmented reality will change the way of human life in the future. With the development of science and technology, VR technology will have great development prospects, as shown in Figure 7.

**Figure 8:**
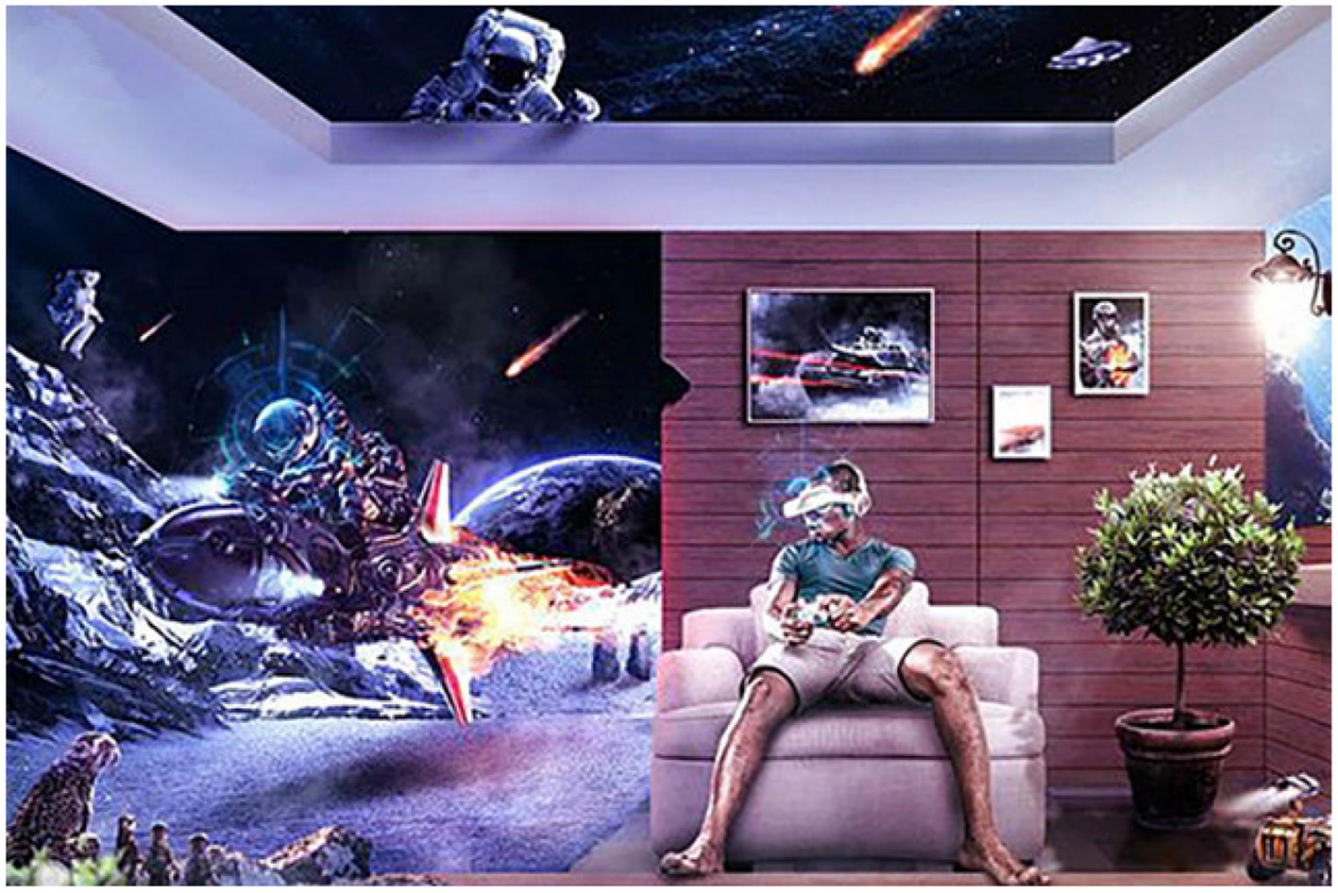
Virtual tourism experience on site (http://alturl.com/g2aeg).

In the stage of data collection of VR tourism products, local cultures need to be substituted into the construction of a virtual tourism scene. The image information collected by the designer as the best “texture” performance of the scenic spot will directly reflect the appearance image of the characteristic landscape, architectural culture, and other material culture of the scenic spot. Whether in the construction of virtual tourist attractions based on two-dimensional images or three-dimensional geometric models, it is necessary to collect the appearance data of tourist destinations in a certain standard and way, as a preliminary presentation of cultural factors. In the process of field data collection, the local intangible culture obtained will be collected as reference materials for future design, as shown in Figure 8.

The more simulation the scene of virtual tourism VR is designed, the easier it is for users to experience the feeling of being on the scene. Therefore, from the perspective of “people (users),” through the investigation and redesign of the real scene of the tourist destination, the “authenticity” of the scene in the virtual tourist attractions should be put in the first place of the construction principles of the virtual tourist attractions. Second, in terms of immersion in interaction, we need to design a natural interaction process to achieve a more “real” interaction behavior, as shown in Table 6.

**Table 6:** Scene interaction process of virtual tourist attractions.

**Serial number**	**Experience feeling**
1	Authenticity
2	Interaction
3	Randomness
4	Fluency
5	Expansibility

**Figure 9:**
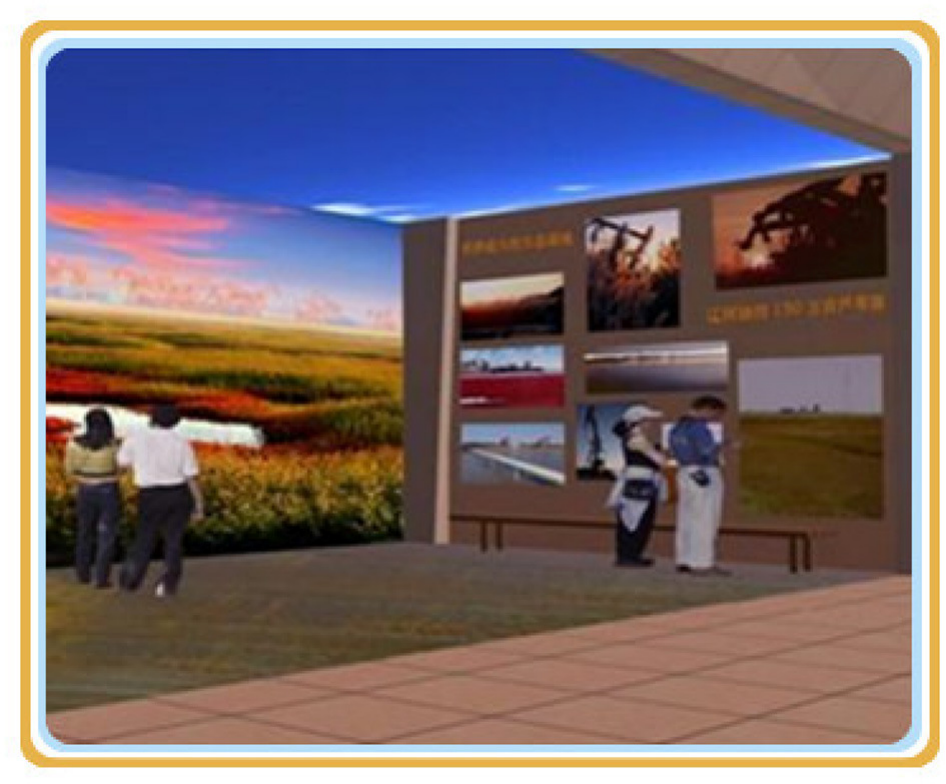
Virtual tourism products (http://alturl.com/aaa7o).

In the stage of scene design in the construction of virtual tourist attractions, processing cultural factors are more important. With the careful embellishment and auxiliary design of cultural factors, the characteristic cultural atmosphere of the tourist destination can be created, so that users can feel the charm of local culture in the scene. In the gradation of scene design, it is difficult to add the idea of developers or designers to the two-dimensional virtual tourist attractions based on image construction design. The purpose of constructing virtual tourist attractions is to enable users to quickly reach the tourist destination in the virtual world for sightseeing activities because the plane images are based on the reality of the tourist destination. In two-dimensional virtual tourist attractions, the appearance features of material culture are directly displayed through two-dimensional images, as shown in Figure 9.

## Conclusion

This study studies the virtual economy effect evaluation of emotional marketing of tourism products based on VR and studies the meaning and algorithm of VR. The analysis of the virtual economy situation of tourism products based on VR shows that the existence and development of VR tourism products are inevitable and historical; however, they cannot completely replace real tourism and become the theme of future tourism mode. Only as an effective supplement to real tourism, they can improve the satisfaction of tourists with real tourism and the quality of the tourism experience. VR tourism products are the products of the combination of tourism and information technology and are the requirements of the development of modern society and the growing tourism demand of human beings. Therefore, VR tourism products have their supporting conditions for existence and development. The characteristics of VR tourism products determine that they will inevitably bring great influence to the current tourism system. It not only effectively solve the lack of transferability of real tourist attractions and the restrictions of real tourism on the leisure and income of tourists but also be affected by the current VR technology and the nature of VR tourism products. At present, VR tourism products can bring tourists only superficial and vulgar tourism experiences.

## Data Availability Statement

The original contributions presented in the study are included in the article/supplementary material, further inquiries can be directed to the corresponding author.

## Ethics Statement

Ethical review and approval was not required for the study on human participants in accordance with the local legislation and institutional requirements. Written informed consent from the participants was not required to participate in this study in accordance with the national legislation and the institutional requirements.

## Author Contributions

MT: experimental designing and editing. SM: experimental process implementing and writing. NX: data collecting and data analysis. All authors contributed to the article and approved the submitted version.

## Conflict of Interest

The authors declare that the research was conducted in the absence of any commercial or financial relationships that could be construed as a potential conflict of interest.

## Publisher's Note

All claims expressed in this article are solely those of the authors and do not necessarily represent those of their affiliated organizations, or those of the publisher, the editors and the reviewers. Any product that may be evaluated in this article, or claim that may be made by its manufacturer, is not guaranteed or endorsed by the publisher.
